# Dynamic Magnetic Resonance Imaging of Whole‐Stomach Motility in Rats

**DOI:** 10.1002/nbm.70138

**Published:** 2025-09-09

**Authors:** Xiaokai Wang, Ulrich M. Scheven, Zhongming Liu

**Affiliations:** ^1^ Biomedical Engineering University of Michigan Ann Arbor Michigan USA; ^2^ Mechanical Engineering University of Michigan Ann Arbor Michigan USA; ^3^ Electrical and Computer Engineering University of Michigan Ann Arbor Michigan USA

**Keywords:** contrast‐enhancement, dynamic MRI, gadolinium, preclinical, rat, stomach

## Abstract

Understanding gastric physiology in rodents is critical for advancing preclinical neurogastroenterology research. However, existing techniques are often invasive, terminal, or limited in resolution. This study aims to develop a non‐invasive, standardized MRI protocol capable of capturing whole‐stomach dynamics in anesthetized rats with high spatiotemporal resolution. Experiments were performed in a 7‐T MRI system. Gadolinium‐doped test meals were prepared to enhance intraluminal contrast in T_1_‐weighted MRI. Based on a modified multi‐slice gradient‐echo sequence, our protocol integrates respiratory gating to minimize motion artifacts, spatial saturation to improve intraluminal contrast, and slice grouping to optimize the trade‐offs between signal‐to‐noise ratio and motion sensitivity. Image acquisition was accelerated using a time‐interleaved k‐space undersampling scheme, with missing data reconstructed through k‐t interpolation. Image quality and gastric motility were quantitatively assessed. The protocol enabled successful imaging of the stomach and visualization of its quasi‐periodic dynamics in anesthetized rats. The gadolinium‐doped meal produced relatively homogeneous intraluminal contrast, allowing clear delineation of gastric anatomy, volume, and motility. The retrospectively reconstructed image exhibited high image quality and yielded reliable estimates of antral contractions, confirming the effectiveness and robustness of the k‐t interpolation method. Estimated antral contraction amplitude and velocity showed minimal deviations from the reference values, whereas contraction frequency estimation remained highly consistent and accurate. Using the accelerated imaging protocol, we imaged the entire stomach and major intestinal regions, acquiring 24 slices with an effective temporal resolution of < 3 s and capturing antral contraction at ~5 cycles per minute. We established an accessible and standardized imaging protocol that encompasses contrast meal preparation, animal handling and training, and a contrast‐enhanced dynamic GI MRI acquisition and reconstruction framework. This protocol provides a comprehensive, robust, non‐invasive tool for studying gastric motility and dysmotility in rodents, offering strong potential to advance preclinical gastrointestinal motility research.

## Graphical abstract

In this paper, we report a standardized, non‐invasive, contrast‐enhanced imaging protocol that encompasses animal handling and training, contrast meal preparation, and a dynamic GI MRI acquisition and reconstruction framework for imaging whole‐stomach dynamics in anesthetized rats.

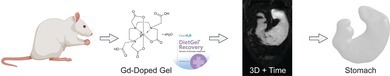

**Abbreviations**
ACS: Auto‐calibration signal
cpm: Cycles per minute
FOV: Field of view
FA: Flip angle
FE: Frequency‐encoding
GI: Gastrointestinal
Gd: Gadolinium
GT: Ground truth
MIP: Maximum intensity projection
PSNR: Peak signal‐to‐noise ratio
PE: Phase‐encoding
R: Reduction factor
RE: Relative error
SEM: Standard error of the mean
SSIM: Structural similarity index

## 1 Introduction

As the gatekeeper for the gastrointestinal (GI) tract, the stomach manages complex motor functions crucial for food intake and digestion [1]. Its proper functioning relies on coordinated movements of its regions: fundus, corpus, antrum, and pylorus [2]. Dysfunction in gastric motility can lead to significant disorders, such as functional dyspepsia and gastroparesis [3, 4]. To prevent and manage such disorders, it is critical to characterize gastric physiology and pathophysiology using techniques that can be translated from preclinical to clinical studies.

Rodents, particularly rats, are widely used in animal studies of GI motility due to their anatomical and physiological similarities to humans [5]. However, current methods for assessing gastric functions in rats differ significantly from those used in clinical practice, creating translational barriers. Traditional preclinical methods, such as marker‐based assays of gastric emptying or food transit [6, 7], are terminal, precluding repeated measurements. Alternative methods involving implanted electrodes or strain gauges [8, 9] are invasive, potentially disrupting physiology or pathophysiology and confounding experimental outcomes.

MRI has emerged as a promising non‐invasive technique for capturing a range of GI functions in both humans and animals [10, 11, 12, 13, 14]. In humans, MRI has been utilized to evaluate gastric accommodation [15, 16, 17], peristalsis [18, 19, 20, 21], secretion [22, 23], intestinal motility [24, 25], and gastric emptying [18, 21]. Relative to human studies, GI MRI applications in preclinical settings remain limited [11, 12, 26, 27]. Previous approaches suffered from either incomplete spatial coverage or compromised speed or resolution, restricting dynamic whole‐stomach imaging of gastric motor function.

Advances in accelerated MRI, utilizing parallel imaging [28, 29, 30], model‐based reconstructions [31, 32, 33, 34, 35], and deep learning [36, 37], have the potential to overcome existing constraints on spatial coverage and spatiotemporal resolution for dynamic GI MRI. These advancements have not yet been thoroughly applied to preclinical gastric MRI, to our knowledge. Herein, we describe a comprehensive dynamic GI MRI protocol specifically designed to capture gastric motility in rats. Our approach leverages k‐t linear predictability to accelerate data acquisition, enabling unprecedented visualization of gastric motility at submillimeter spatial resolution and high temporal fidelity with a whole‐stomach field of view (FOV). Our MRI acquisition and reconstruction method, combined with optimized protocols for animal handling and contrast‐labeled meal preparation, establishes a standardized, non‐invasive imaging platform ideal for preclinical studies of GI motility in health and disease.

## 2 Methods and Materials

### 2.1 Animals

Fourteen Sprague–Dawley rats (male, 230–430 g, Envigo, Indiana, United States) were used for this study. All experimental procedures were approved by the Unit for Laboratory Animal Medicine and the Institutional Animal Care and Use Committee at the University of Michigan. Rats were housed under controlled conditions (temperature 68–79 °F, relative humidity 30–70%) and a 12:12 h dark–light cycle.

Rats were provided with a standardized test meal, either ingested voluntarily (*n* = 12) or administered through intragastric gavage (*n* = 2), 10–30 min prior to MR imaging. To facilitate the voluntary ingestion, 12 rats underwent a 7‐day diet training protocol before the imaging experiment [11, 38, 39]. Briefly, during the first 2 days, rats had ad libitum access to both regular chow and diet gel (DietGel Recovery, ClearH2O, ME, USA) until 6 p.m. on the second day. From day 3 to day 7, rats only had ad libitum access to the diet gel from 12 p.m. to 6 p.m., with no food provided outside these hours. Water remained available at all times. Following this training, rats were able to consume ~5 g of the test meal within 30 min after being fasted overnight for 18 h. For intragastric administration, rats were acclimated to gentle but firm handling and restraining for at least 2 days before the imaging experiment. They were given a total of ~3.4 g of test meal through intragastric gavage in up to three doses within 10 min. We retrospectively simulated and evaluated practical considerations in two rats following intragastric gavage, and tested the accelerated acquisition in all 14 rats.

### 2.2 Contrast Agent and Test Meal

**FIGURE 1:**
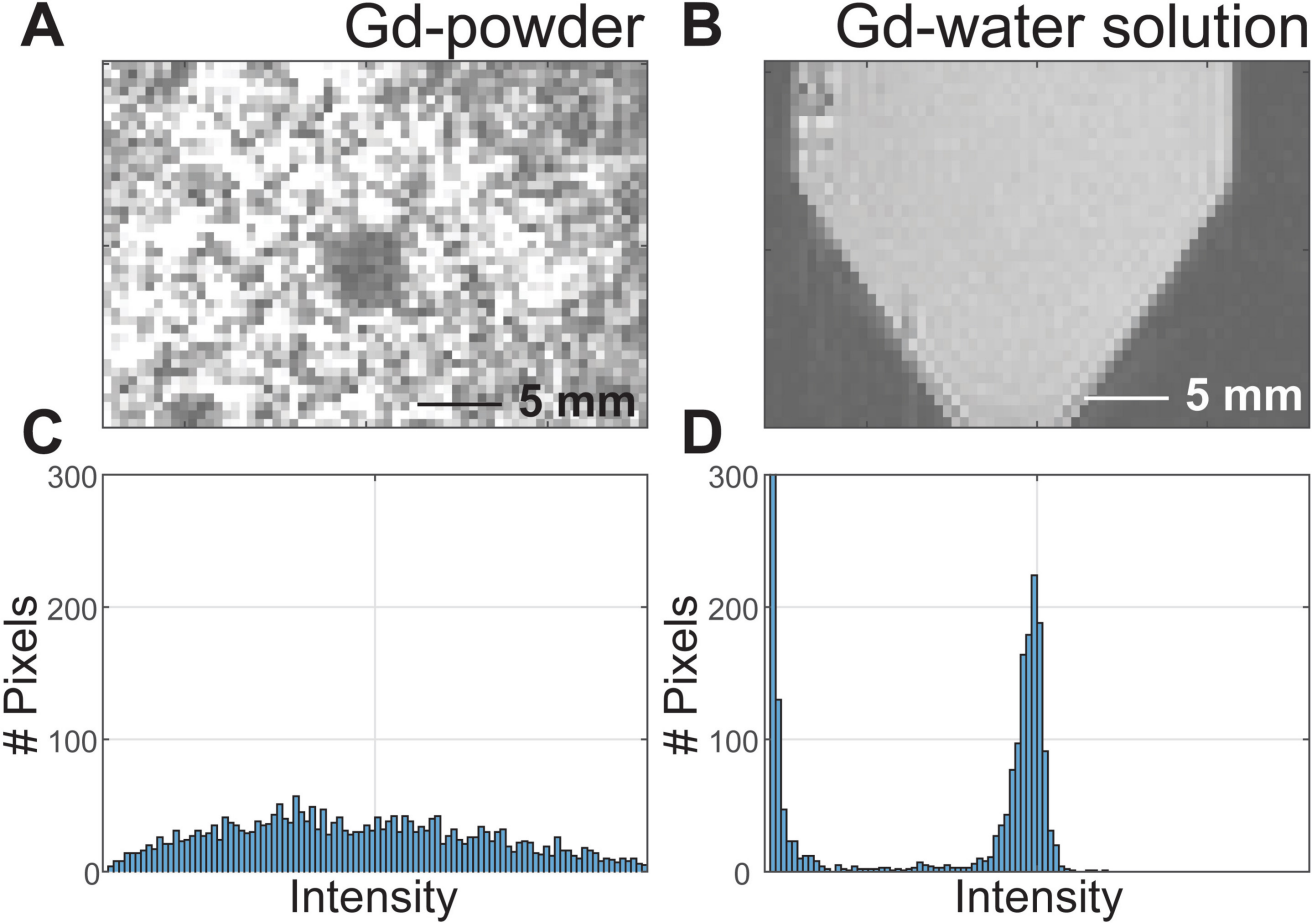
Characterization of Gd‐doped meals. (A) Diet gel is mixed with Gd‐DTPA in powder form (Gd‐powder). (B) Diet gel is mixed with Gd‐DTPA dissolved in water (Gd‐water solution). Correspondingly, the pixel intensity distribution of images with Gd‐powder and Gd‐water solution is in (C) and (D).

**FIGURE 2:**
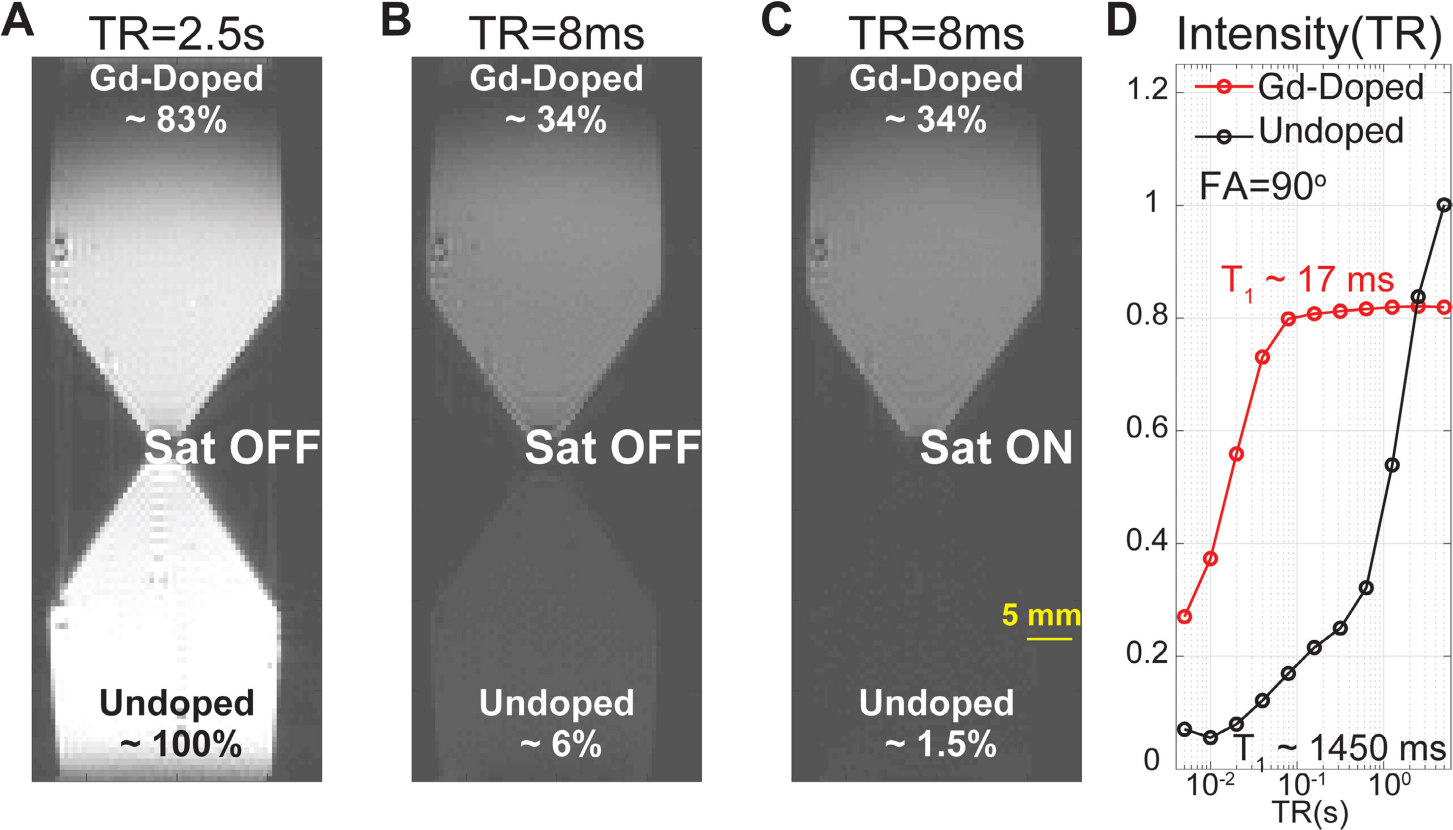
Gradient echo images of undoped and Gd‐doped test meals, for different repetition time (TR), with and without spatial saturation. (A) TR = 2.5 s, (B) TR = 8 ms, (C) TR = 8 ms with spatially non‐selective saturation. (D) Pixel intensity of undoped and Gd‐doped meals, as a function of TR. Intensities are all normalized to the signal of the undoped meals at long TR. Other key imaging parameters include TE 0.99 ms, FA 90°, matrix size 128 × 64, resolution 0.625 mm × 0.5 mm, thickness 2 mm.

The test meal was a homogeneous and semi‐solid mixture of diet gel and gadolinium (Gd)‐ DTPA (#381667, Sigma Aldrich, Missouri, United States). Specifically, the gel provided 112.4 kcal per 100 g, including 71–75% water, 23.1% carbohydrates, 1.9% fat, and 0.6% protein. We made a 1 mL, 182 mM Gd‐water solution with a probe sonicator (Q700, Qsonica, Connecticut, United States), and further mixed it with 25 mL of heated, liquified gel to prepare a 7 mM Gd‐doped mixture. The mixture was vortexed and cooled overnight to yield a uniform, solidified test meal (Figure 1) with a short T_1_ relaxation time (~17 ms at 7 T) (Figure 2).

### 2.3 MRI Acquisition

**FIGURE 3:**
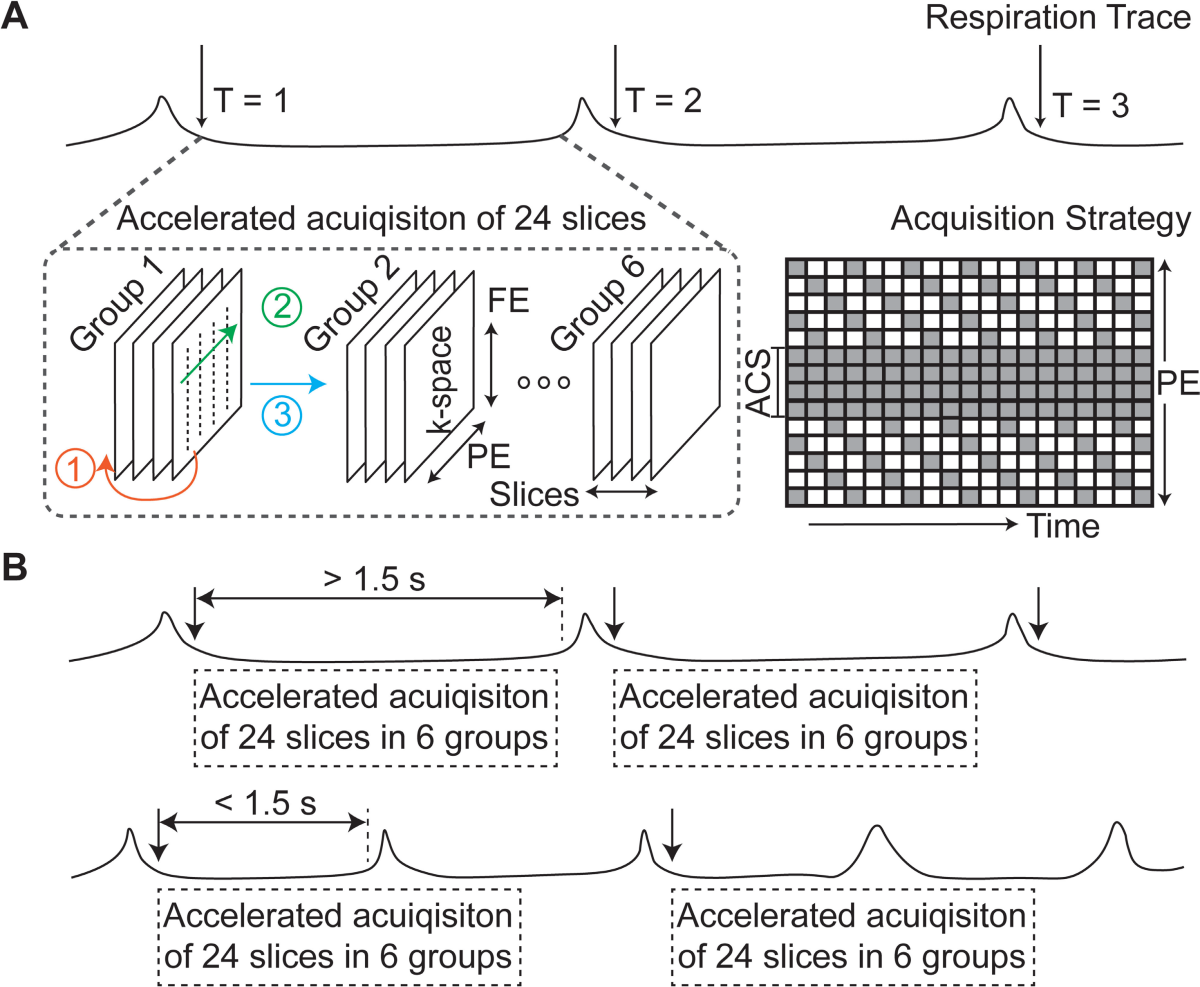
Schematic acquisition strategy. (A) Respiration‐triggered accelerated acquisition. The respiratory signal of anesthetized rats was used to trigger the image acquisition. Each respiration cycle is characterized by a short spike in pressure, due to rapid respiratory motion at that time (inhalation), followed by a longer and reasonably quiescent interval (slow exhalation). Arrows at T = 1, T = 2, and T = 3 mark the gating signal at the beginning of a complete stomach image acquisition. Following the gating signal, 24 slices are acquired in 6 consecutive groups (packets) of 4 adjacent slices, as described in the text. Colored numbers indicate the order of the loop structure, from slice dimension, phase encoding (PE) direction, and groups. The k‐space sampling pattern is plotted along PE (vertical axis) and time (horizontal) dimensions. The sparse k‐space sampling pattern repeats every three acquisitions in this schematic diagram, representing a reduction factor of three. Each box is one line of frequency encoding (FE). Gray box: acquired PEs, white box: skipped PEs, ACS: autocalibration signals. (B) Two acquisition schemes with the respiration cycle longer or shorter than 1.5 s (the time it takes to acquire 24 slices with the accelerated acquisition schemes).

Imaging was performed in a 7‐T horizontal‐bore small‐animal MRI system (7 T/310/ASR, Agilent Technologies, California, United States) equipped with a gradient set (inner diameter 120 mm; strength 400 mT/m) and a single‐channel volume transmit and receive ^1^H RF coil (inner diameter 60 mm). Rats were anesthetized [38, 39] and maintained stable physiology (respiration 30–60 cycles per min (cpm), heart rate 220–310 beats per min, body temperature ~37 °C), monitored via an MRI‐compatible system (Model 1030, SA II, New York, United States). The respiratory signal was recorded with a pressure‐sensitive pillow placed under the animals. Figure 3 depicts schematically a typical respiration trace of rats under anesthesia. The image acquisition was triggered by the respiratory signal and confined to the quiescent phase (slow exhalation) to minimize motion artifacts. With a respiration rate of 30–60 cpm, the time window for each MRI acquisition is 1–2 s per respiration cycle. The proposed accelerated imaging protocol is detailed below. Reference scans used for retrospective simulation or comparison are described in context.

Images were acquired using a hybrid multi‐slice gradient‐echo sequence with 24 slices (1.5 mm thickness) grouped into six packets of four adjacent slices each and acquired consecutively (Figure 3). This scheme was chosen to ensure signal intensity by using an adequate TR for magnetization recovery, while also minimizing motion artifacts by limiting the time window during which each slice was acquired. Each respiration‐triggered acquisition scanned slices sequentially from the ventral to the dorsal side. Acquisition parameters included TR = 10.6 ms, TE = 1.6 ms, flip angle (FA) = 25°, FOV = 64 × 42 mm^2^, matrix size = 128 × 84, and nominal in‐plane resolution = 0.5 × 0.5 mm^2^.

Spatially non‐selective saturation pulses were applied immediately before each packet of slices to suppress signals from surrounding tissues and flowing blood. This approach enhanced gastric contrast by leveraging the rapid T_1_ recovery of Gd‐doped contents (T_1_ ~ 17 ms) compared to the much slower recovery of surrounding tissues (T_1_ > 800 ms), while reducing motion‐related and aliasing artifacts. Given our effective recovery time of ~127 ms (defined as the interval between the saturation pulse and the gradient echo at the center of k‐space), the longitudinal magnetization of gastric content fully recovered, while signals from surrounding tissues recovered only marginally (< 20%) (Figure 2).

Cartesian acquisition was accelerated using a time‐interleaved undersampling scheme in k‐space. As illustrated in Figure 3A, phase‐encoding (PE) lines (bandwidth 100 kHz) were partially sampled at consecutive time points, alternating skipped lines to achieve a reduction factor (R) of 3 at the peripheral k‐space (12 lines), while the auto‐calibration signal (ACS, 12 lines) was fully sampled at the k‐space center. This sampling scheme resulted in a total acquisition time of roughly 1.5 s per volume (24 slices). Upon the respiration trigger, the acquisition starts and continues until the volume is finished (Figure 3B). Specifically, in cases where the quiescent phase of the respiratory signal was shorter than 1.5 s (respiratory rate > 40 cpm), the acquisition of a volume would continuously span two or more respiratory cycles. This resulted in a maximal effective temporal resolution of 3 s. Eventually, the proposed accelerated acquisition protocol balanced the spatial and temporal resolution, enabling full stomach coverage.

### 2.4 MRI Reconstruction

**FIGURE 4:**
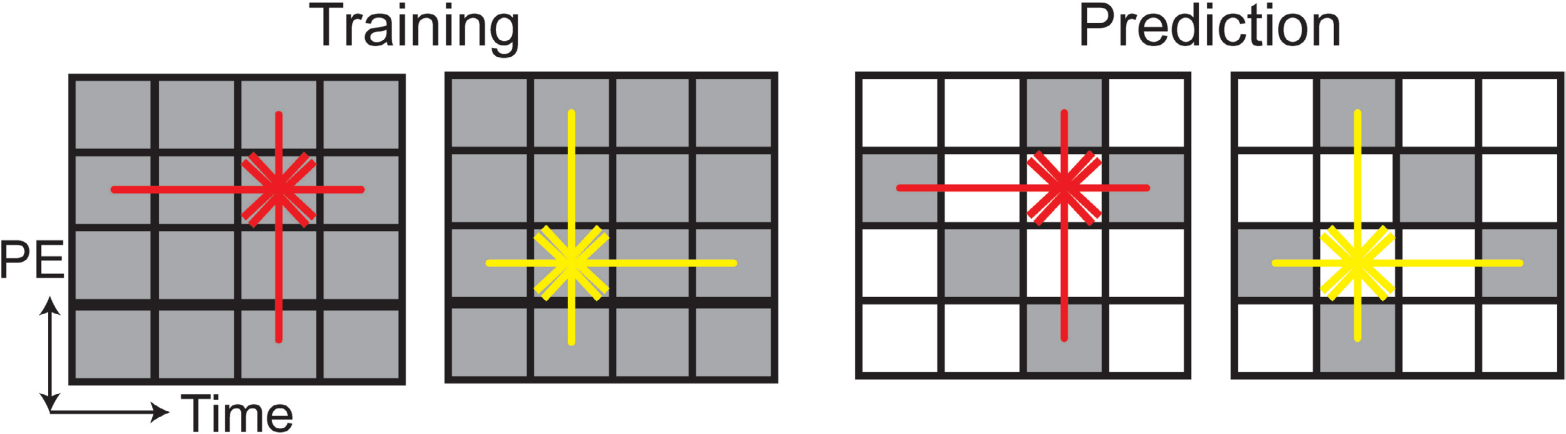
Interpolation kernels for image reconstruction. Take the reduction factor, R = 3, as an example. Two kernels (red and yellow) with distinct shapes were used. During training, acquired PEs at the central k‐space (ACS, gray) were used to train the kernel weights to predict the other, following the colored arrows. During prediction, skipped PEs (white) at the peripheral k‐space were estimated using the trained kernels, following the colored arrows.

Reconstruction leveraged linear predictability [40] in k‐t space to interpolate the undersampled k‐space data (Figure 4), adapting principles similar to k‐t GRAPPA [41] for a single‐coil scenario. Our justification for linear predictability relied on two key assumptions. First, the spatially uniform Gd‐doped gastric contents created regions of piecewise homogeneity in the image space, effectively acting as spatial multipliers analogous to coil sensitivity profiles in parallel imaging, thereby corresponding to convolution kernels in k‐space. Second, gastric motility exhibited quasi‐periodic motion (~5 cpm), characterized by narrow‐band frequency components, thus justifying the assumption of time‐invariant convolution kernels for temporal interpolation. With these considerations, k‐t convolutional interpolation kernels derived from fully sampled ACS (12 central k‐space lines) could be used to accurately estimate the missing peripheral samples despite an undersampling factor of R = 3 or higher. Specifically, we estimated the kernel weights by fitting with ACS and minimizing the least square errors. No regularizers were used in estimating kernels. This approach facilitated robust reconstruction, yielding high‐resolution dynamic images with clear delineation of gastric structures and motility.

### 2.5 Validation and Evaluation

Validation of the reconstruction approach and evaluation of practical choices were performed with retrospective simulations and accelerated acquisitions. Retrospective simulations involved applying various reduction factors (R = 2, 3, 4 through 11) and numbers of auto‐calibration signal lines (ACS = 12 and 24) to fully sampled reference datasets (four slices with spatial saturation, TR = 10 ms, TE = 1.4 ms, FA = 25°, acquisition time = 0.9 s per image volume). Reconstruction performance was evaluated by computing quantitative metrics relative to the fully sampled reference images, including relative error (RE) [42], structural similarity index (SSIM) [43], and peak signal‐to‐noise ratio (PSNR). Before calculating these metrics, images were normalized to [0, 1]. In addition, gastric motility metrics, including contraction amplitude, frequency, and propagation velocity, were extracted from the reconstructed images and compared against fully sampled reference data using the procedures described elsewhere [11]. Briefly, the longitudinal axis of the antrum was first identified to analyze cross‐sectional areas perpendicular to the axis. From the area‐time series, peaks (stomach relaxation) and valleys (stomach contractions) were detected. Contraction amplitude was defined as the occlusion ratio caused by contractions, estimated using the peak‐to‐valley difference normalized by the peak. Frequency was determined as the dominant frequency via the Fourier transform of the area‐time series. Propagation velocity was calculated by dividing the distance between cross‐sections by the time delay it took the contraction wave to travel between them. Paired *t* tests were performed to evaluate the statistical significance of the differences in motility measures (a significance level of alpha = 0.05).

The proposed accelerated imaging protocol was tested in all anesthetized rats. Reconstructed images were assessed for their ability to accurately depict gastric dynamics and anatomy. Gastric motility metrics were evaluated using an approach adapted from [11] and compared with the normal range reported in the same study. Unlike the original method, which used only four slices, our accelerated acquisition covered the entire stomach, allowing the use of as many slices as needed to fully cover the antrum for a more accurate gastric motility assessment. Gastric volumes measured from time‐averaged dynamic images were quantitatively compared with anatomical reference scans (same slice thickness, no spatial saturation, respiration gated, 24 slices with two adjacent slices per packet, TR = 5.0 ms, TE = 1.4 ms, FA = 20°, NEX = 4, FOV = 64 mm × 64 mm, matrix size = 128 × 128, total acquisition time = ~38 s) obtained immediately before or after dynamic acquisitions. Pearson correlation was calculated to statistically evaluate the agreement between these volumetric measures (a significance level of alpha = 0.05).

## 3 Results

### 3.1 Respiratory Gating and Motion Artifact Reduction

**FIGURE 5:**
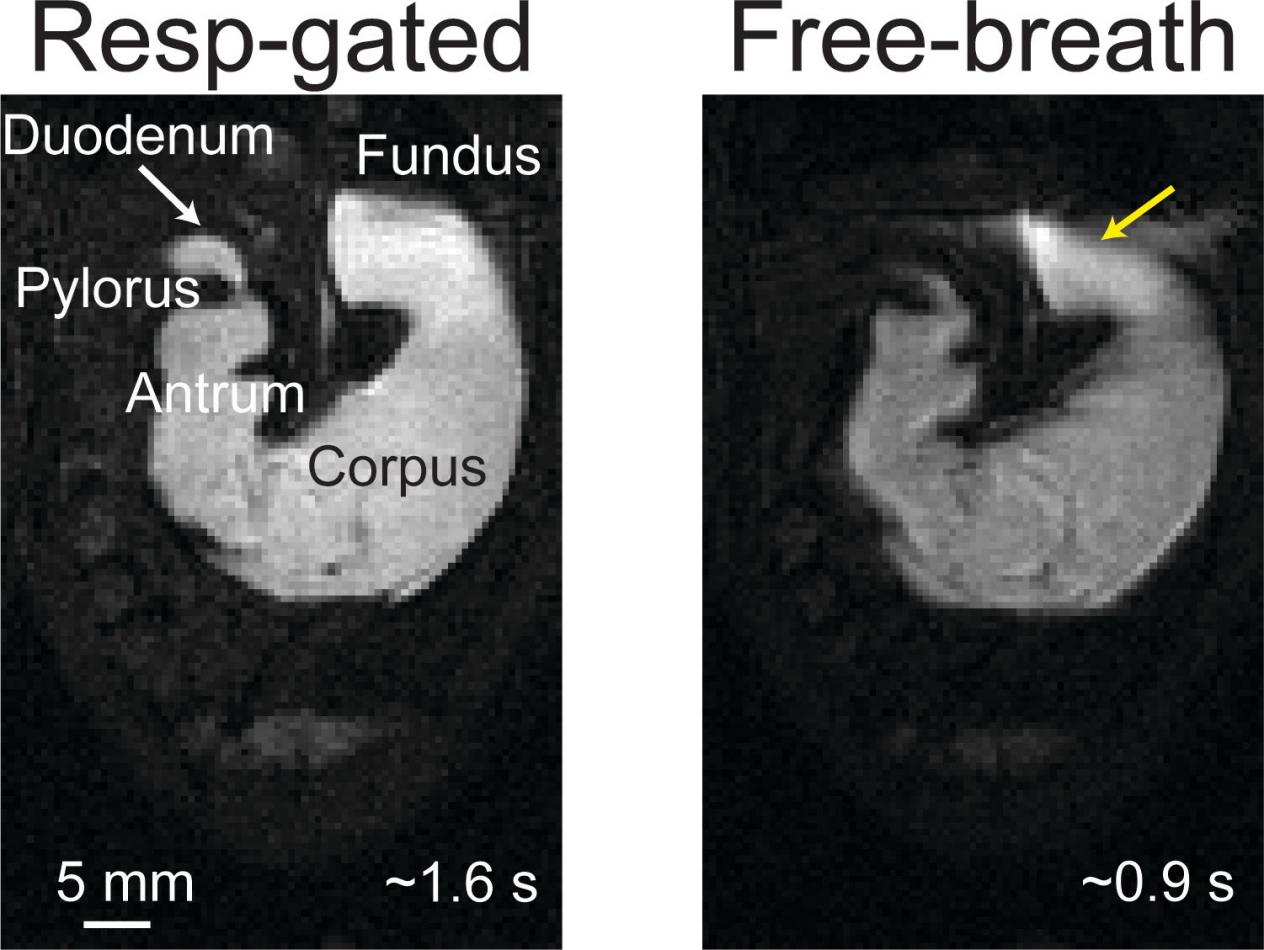
Effects of respiration gating. A representative slice from fully sampled scans with (left) or without respiratory gating (right). The yellow arrow points to the fundus, where respiratory movements cause distortion. The white arrow points to the duodenum. The representative Images acquired with respiratory gating took ~1.6 s per volume, whereas images acquired without respiratory gating took ~0.9 s per volume.

Respiratory gating significantly reduced motion artifacts associated with respiration and ensured stable, repeated acquisitions at consistent respiratory phases. Representative images (four slices, fully sampled, TR = 10 ms, TE = 1.4 ms, FA = 25°) acquired with respiratory gating demonstrated clearer delineation of gastric geometry, particularly near the diaphragm and pylorus, compared to images acquired without gating (Figure 5). Although sampling intervals were constrained by the duration of the respiratory cycle (typically 1–2 s per cycle), the resulting sampling rate (0.5–1 Hz) remained sufficient to capture gastric motility dynamics (~5 cpm).

### 3.2 Effects of Spatially Non‐Selective Saturation Pulses

**FIGURE 6:**
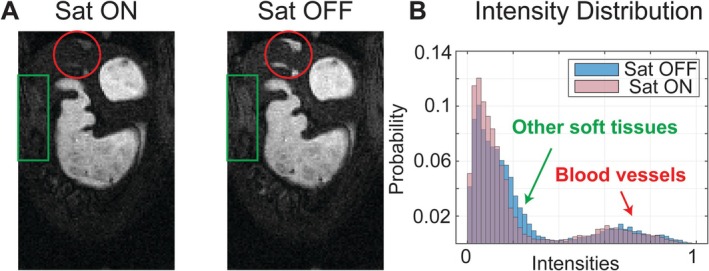
Effects of spatially non‐selective saturation. (A) Fully sampled images with (left) or without spatially non‐selective saturation pulses (right). (B) The distribution of normalized pixel intensities ([0 1]) from both images. Green arrows and boxes highlight other soft tissues and aliasing artifacts. Red arrows and boxes highlight blood vessels.

Spatially non‐selective saturation pulses enhanced gastric image quality by suppressing signals from surrounding tissues and flowing blood. Comparative images acquired with and without saturation pulses (Figure 6) demonstrated improved intraluminal contrast due to the rapid T_1_ recovery of Gd‐doped gastric contents (~17 ms) compared to the much slower T1 recovery of surrounding tissues (> 800 ms). Importantly, saturation pulses had a negligible impact on the Gd‐doped test meal (Figure 2), and similarly for the intraluminal signal. Figure 6 also highlights how saturation pulses effectively reduced bright blood signals and peripheral aliasing artifacts, particularly near major blood vessels and tissue boundaries. These improvements substantially increased the clarity of gastric structures, simplifying image segmentation and enhancing interpretability.

### 3.3 Retrospective Simulation and Reconstruction

**FIGURE 7:**
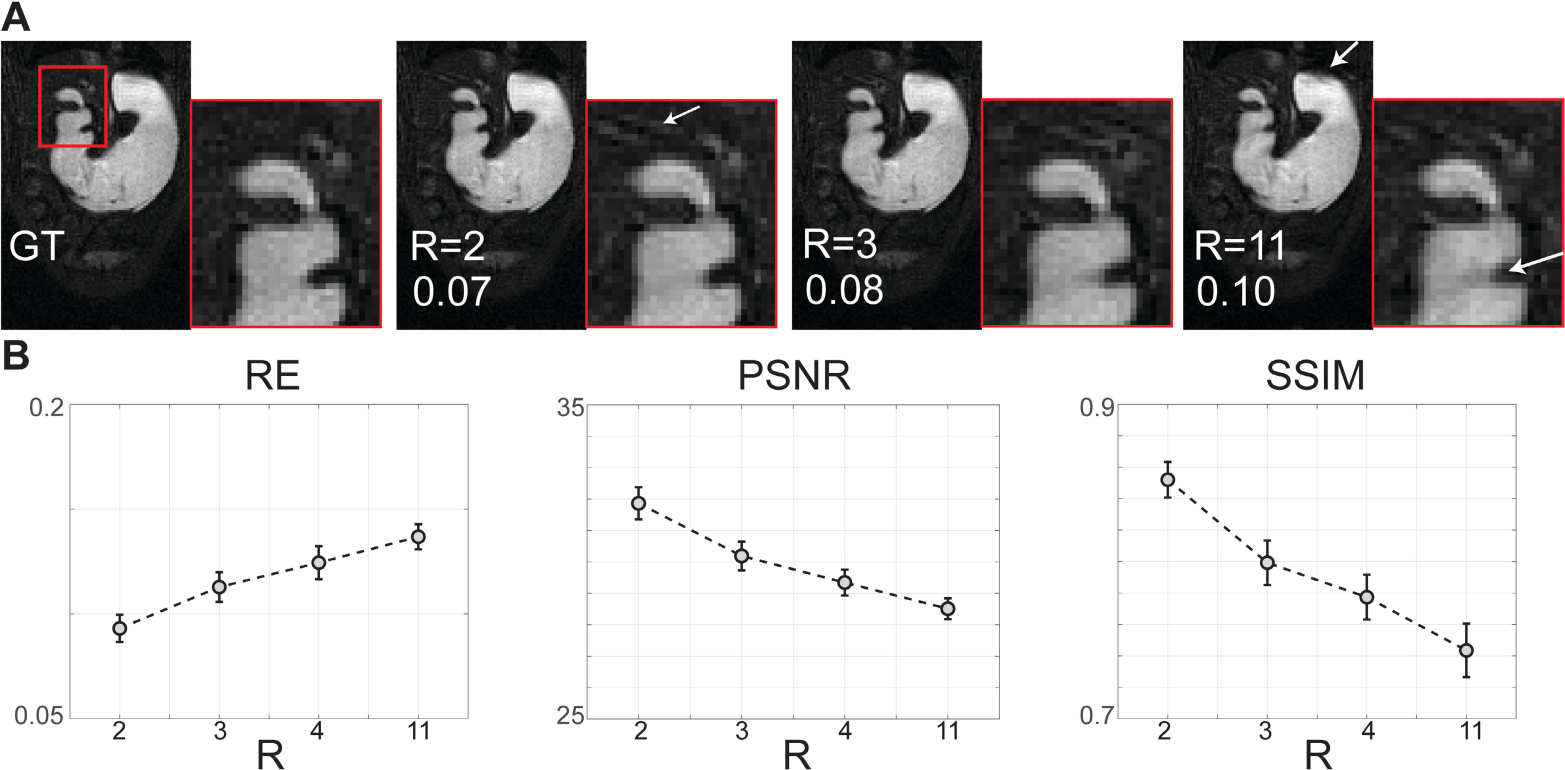
Effects of the reduction factor (R) in retrospective simulations. (A) Ground truth (GT) reference image reconstructed with fully sampled k‐space, and the images reconstructed from retrospectively down‐sampled k‐space with R = 2, R = 3, and R = 11 (ACS = 12). Numbers on images are the relative errors (RE) between each reconstructed image and the GT image. The distal antrum, pylorus, and duodenum, highlighted by the red box, are zoomed in further. White arrows point to the artifacts. (B) The RE, peak signal‐to‐noise ratio (PSNR), and structural similarity index (SSIM) between GT and each reconstructed image with different R. The RE is calculated by the L2 norm of the intensity differences between two vectorized images divided by the L2 norm of the reference.

**FIGURE 8:**
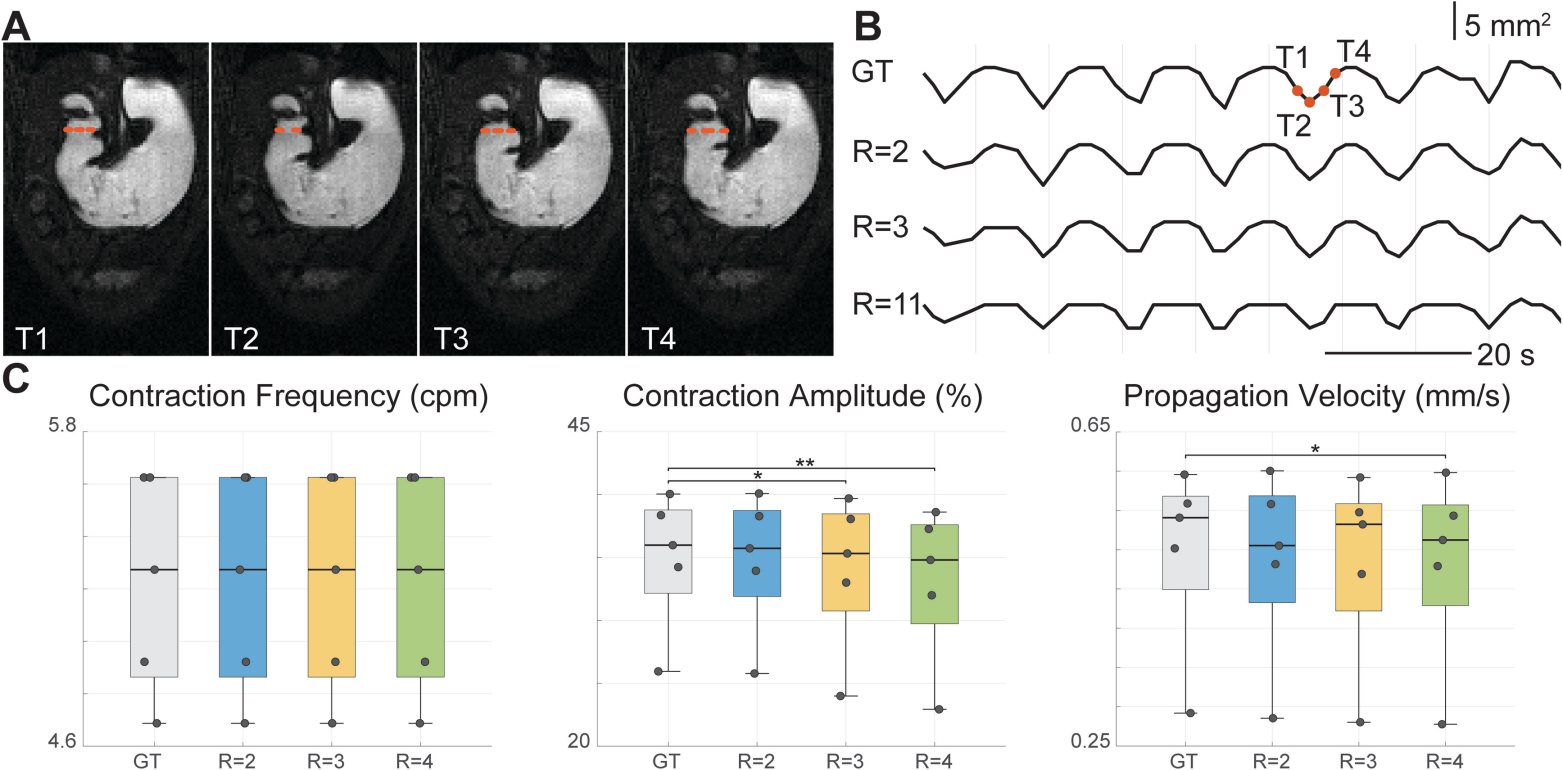
Effects of the reduction factor (R) in retrospective simulations on antral contraction quantification. (A) A time series of fully sampled dynamic MR images from a representative slice, as the ground truth (GT) reference images (T1–T4). (B) A time series of cross‐section areas, measured from GT images and images reconstructed from retrospectively down‐sampled k‐space with R = 2, R = 3, and R = 11 and ACS = 12. Red dashed lines in (A) show the cross‐section plotted in (B). (C) Quantified contraction frequency, amplitude, and propagation velocity from GT, and images reconstructed from retrospectively down‐sampled k‐space with R = 2, R = 3, and R = 4 (ACS = 12). Asterisk signs indicate statistical significance. *p < 0.05, **p < 0.01.

Retrospective reconstructions from simulated undersampling scenarios (reduction factors R = 2 through 11; ACS = 12 and 24) confirmed the effectiveness and robustness of convolution‐kernel‐based k‐t interpolation (Figure 4). Minimal degradation in image quality was found at moderate undersampling (R = 2, 3). At higher undersampling (R = 11), noticeable increases in image blurring and reconstruction artifacts were observed (Figure 7 and Supplementary Table S1). Quantitative evaluations demonstrated good reconstruction fidelity, with RE remaining low even at higher reduction factors. SSIM and PSNR metrics indicated similar trends (Figure 7 and Supplementary Table S1). Gastric motility dynamics were successfully captured from retrospective reconstructions. Quantitative gastric motility metrics, such as contraction frequency, remained robustly measurable across the undersampling scenarios tested (Figure 8 and Supplementary Table S2), while contraction amplitude and velocity were only mildly affected despite the statistically significant differences (Figure 8 and Supplementary Table S2).

### 3.4 Accelerated Acquisition and Reconstruction

**FIGURE 9:**
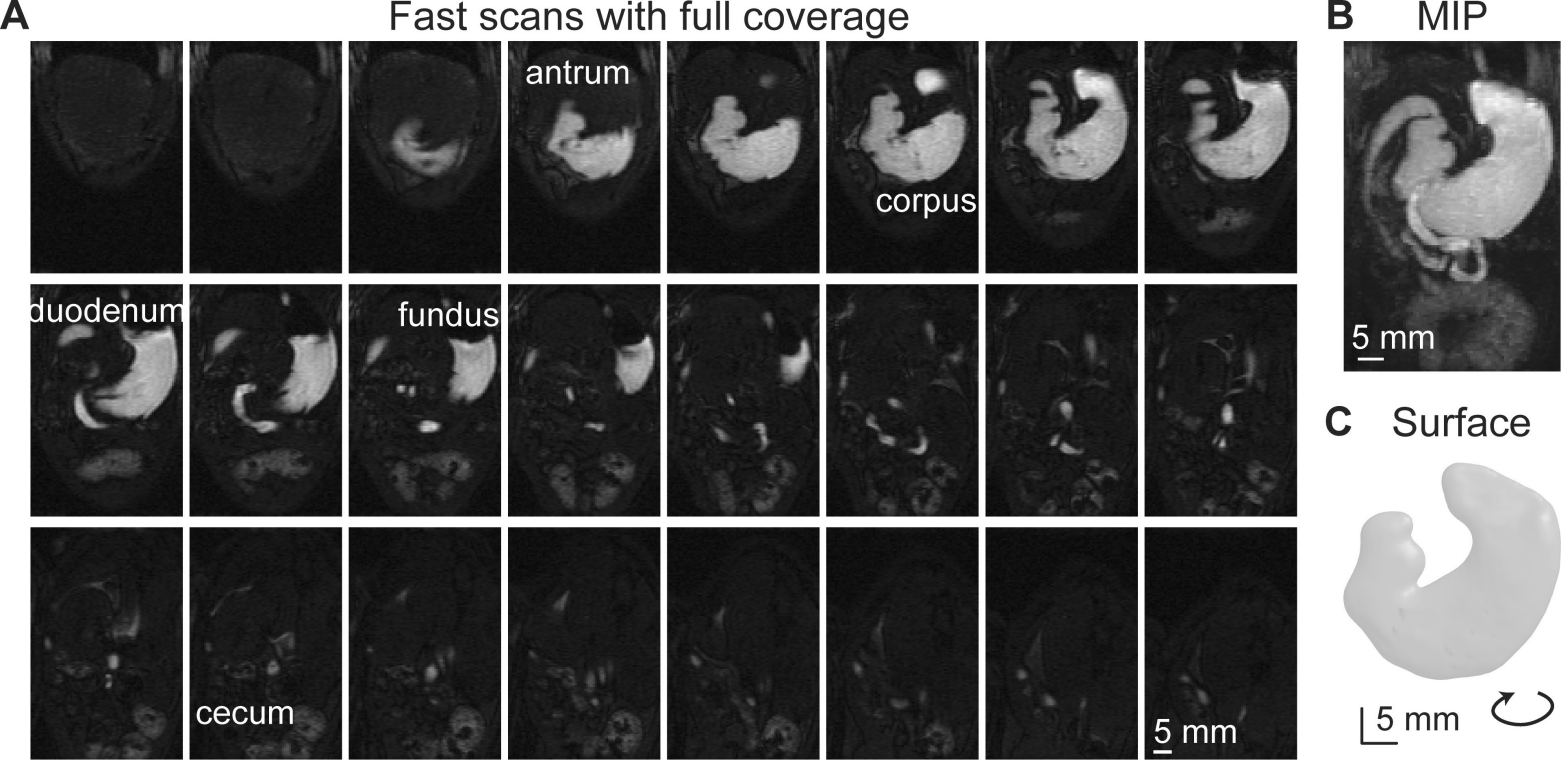
A representative image volume of 24 slices from accelerated acquisitions covers the entire stomach and small intestines. (A) In this representative example, all 24 slices were acquired over roughly 1.5 s, approximately one respiration cycle. (B) The maximum intensity projection (MIP) across 24 slices. (C) The corresponding 3D stomach surface model. The surface model is rotated slightly for a better visualization.

**FIGURE 10:**
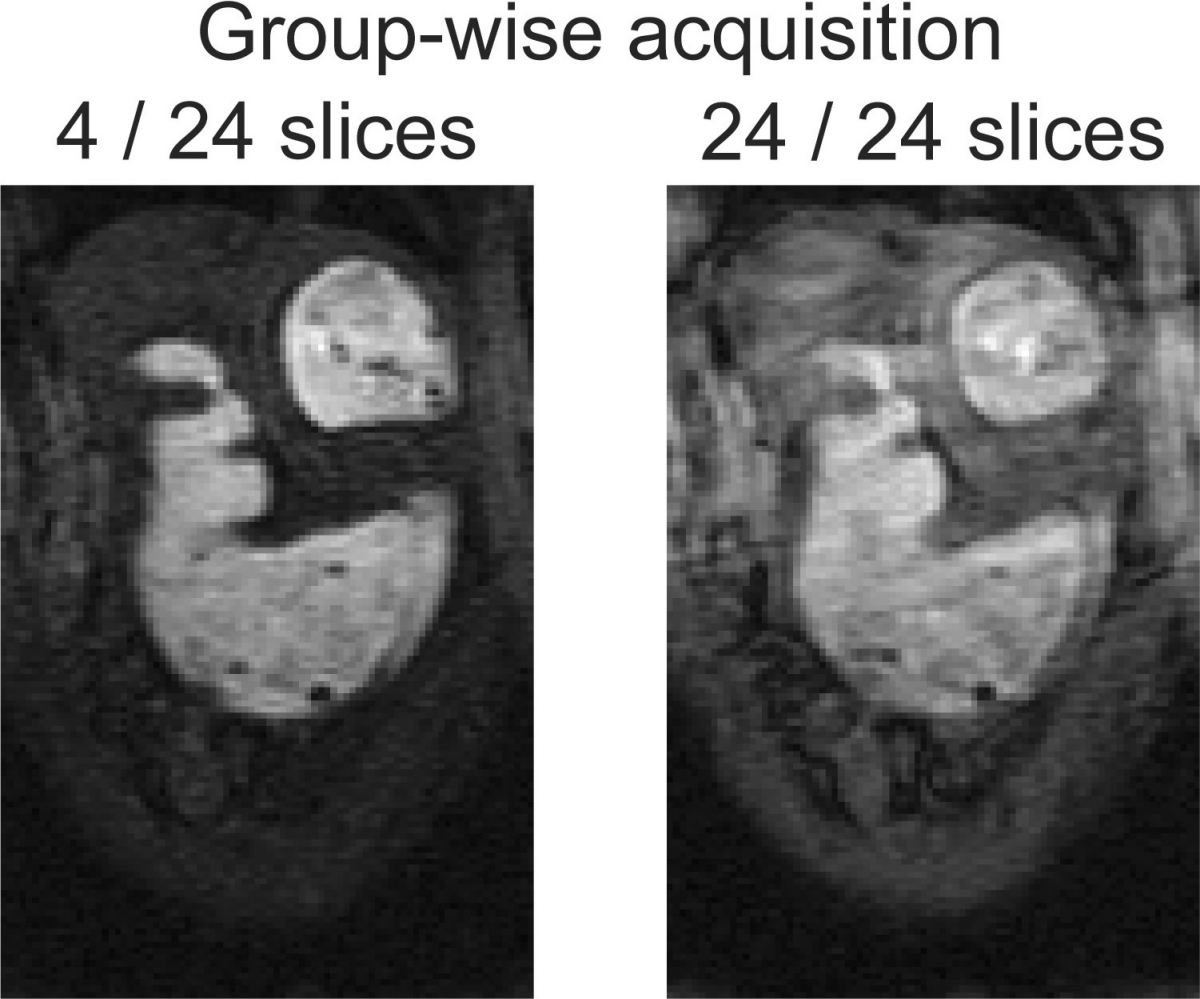
Effects of slice grouping. Left: A slice acquired within a packet of 4 adjacent slices, having an acquisition time of 256 ms per packet. Right: A comparable slice acquired within a group of 24 slices, with an acquisition time of 1440 ms. A shorter acquisition time, compared to a respiration cycle of 1–2 s, reduces motion artifacts. These representative images were acquired without respiration gating.

The accelerated acquisition successfully achieved comprehensive stomach coverage, capturing dynamic gastric motility at high spatial and temporal resolutions (Figure 9). Each dataset was acquired rapidly (effective temporal resolution < 3 s), clearly delineating GI anatomical regions such as the fundus, corpus, antrum, pylorus, and duodenum (Figure 9A). Intraluminal signal intensity was lower in the corpus and antrum compared to the fundus. The slice grouping scheme effectively balanced signal intensity and motion sensitivity. Compared to standard multi‐slice gradient echo sequences without slice grouping, images with the slice grouping scheme were significantly less susceptible to motion artifacts, especially at high respiratory rates (e.g., > 45 cpm). A representative example (Figure 10) illustrates that acquiring slices in packets results in a short time window (~256 ms) for acquiring each packet and eliminates respiratory motion artifacts. Conversely, standard acquisitions without slice grouping used a longer time window (~1440 ms) to fill in k‐space, coinciding with respiratory cycles and degrading image quality. Additionally, given a longer TR at the same FA, images acquired without slice grouping also show poorer contrast with brighter structures surrounding the GI tract. Maximum intensity projections (MIP) (Figure 9B) and the reconstructed 3D surface model (Figure 9C) further demonstrated the high fidelity and detailed visualization of gastric morphology achieved by the proposed accelerated imaging protocol.

The reconstructions of the accelerated acquisitions accurately depict gastric dynamics and anatomy. Gastric contractions and their propagation are clearly visible along both the lesser and greater curvatures (Figure 9). Also, see dynamic stomach motions in Supplementary Videos S1 and S2. For all rats (*n* = 14), the stomach contractions have an amplitude of 23.1% (mean) ± 1.5% (standard error of the mean, SEM), a frequency of 5.3 ± 0.1 cpm, and a speed of 0.56 ± 0.03 mm/s. Gastric volume measurements (*n* = 12) derived from dynamic acquisitions (5.2 ± 0.2 mL) exhibited strong agreement with the reference slow, anatomical scans (5.5 ± 0.2 mL) acquired in close temporal proximity (*r* = 0.98, *p* < 0.001). These comprehensive visualizations and quantifications confirm the effectiveness of the proposed dynamic MRI approach for preclinical gastric motility studies.

## 4 Discussion

In this paper, we describe an optimized dynamic MRI protocol and demonstrate its effectiveness for capturing whole‐stomach motility in anesthetized rats. Although our method is an adaptation of existing methods rather than a radical innovation, its systematic integration of Gd‐labeled meal, respiratory gating, slice grouping, and k‐t undersampling and interpolation addresses several practical hurdles commonly encountered in preclinical GI MRI. Our design elements, evaluated individually and collectively, substantially improve signal uniformity, reduce motion artifacts, and yield robust measurements of gastric volumes, contraction amplitude, frequency, and velocity. The high‐contrast images allow for clear delineation and easy segmentation of gastric contents, while the short scanning time (effective temporal resolution < 3 s for the entire stomach) preserves sufficient temporal resolution to track gastric contractions and coordination.

As a key advantage, this protocol achieves large spatial coverage and high temporal resolution, while maintaining the high spatial resolution necessary to resolve fine structures, such as the pylorus. In order to achieve high scanning speed, previous rat MRI studies only capture a part of the stomach [11], lacking the ability to evaluate motility across the entire stomach. Here, in our study, the proposed protocol combines full‐stomach coverage, submillimeter resolution, and an effective temporal resolution of < 3 s. It enables accurate assessment of gastric geometry, volume, and motility and makes it possible to track gastric wall motion and uncover regional differences in contractile activity. Compared with the slower anatomical scans (~38 s), it captures gastric geometry with contractions clearly resolved over time, avoiding motion blur from propagating contractions and improving volume estimation accuracy. Furthermore, the derived contraction amplitude, frequency, and propagation velocity are comparable to the previously reported normal ranges in rats [11], supporting the validity of the proposed protocol for quantifying gastric motility. Adding concentrated Gd‐DTPA to the test meal provides strong intraluminal contrast against surrounding tissues and organs, even when dilution within the stomach occurs due to gastric secretion. Saturation pulses selectively suppress extraluminal signals, exploiting the significant T_1_ difference between Gd‐doped contents (~17 ms) and tissues (>800 ms). In addition, respiratory gating aligns acquisition, minimizing image blurring and motion artifacts. The resultant dataset can report anatomical, volumetric, and motility metrics at global, regional, and subregional levels, enabling detailed evaluation and mapping of gastric motor function [21, 38].

Our study extends the practical applicability of k‐t interpolation. Single‐coil GI MRI is challenging because typical parallel imaging frameworks require multiple coils bearing different spatial sensitivities. In our study, when filled with the Gd‐doped test meal, the lumen of the stomach is homogeneous and brighter than the surrounding tissues and organs, rendering the image as a combination of two separate spatial profiles: one with high intensity (inside the lumen) and the other with low intensity (outside the lumen). The spatial profile, equivalently working as an effective “sensitivity” pattern as in a multi‐coil scenario, supports the assumption of a shift‐invariant convolutional kernel in k‐space. Further, the rhythmic nature of stomach movements with a narrow dominant frequency supports linear predictability over time. A finite impulse response function can be used to accurately pick up the rhythmic dynamics, or equivalently, a time‐invariant convolutional kernel. In summary, with a single coil, the unique properties of contrast‐enhanced GI MRI allow the use of the k‐t interpolation for imaging stomach movements. This strategy allows for accelerated data acquisition while retaining motion details. Although this method is similar to k‐t GRAPPA, initially introduced for dynamic cardiac imaging [41], its application in small‐animal GI MRI is our unique contribution, adding a compelling dimension to enhance the practicality of preclinical imaging of gastric motility.

By offering a non‐invasive readout of gastric motility patterns, our protocol has broad implications. It can be used to test new prokinetic agents or neuromodulatory drugs in rodent models [44, 45], where effects can be monitored in real time. Researchers exploring vagus nerve stimulation, optogenetic or chemogenetic stimulation, or target lesioning can likewise quantify how neural control or ablation affects gastric motility. Our method can be used in disease models to interrogate pathophysiology in GI disorders, such as gastroparesis, and provide mechanistic insights into how various gastric regions and their motor events contribute to delayed gastric emptying. Our method can also provide a deeper understanding of gastric physiology. By varying the composition of ingested test meals (e.g., different nutrient profiles or meal volume) or manipulating gastric pH and secretions, their effects become quantifiable through the volumetric and dynamic measures obtained with gastric MRI. Due to the non‐invasive nature of MRI, it is plausible to collect repeated, longitudinal data in the same animal, benefiting chronic studies and supporting robust experimental designs as well as better translation to humans.

Applying advanced coil designs, parallel imaging, or compressed sensing may further shorten acquisition time or enhance spatial resolution, aiding studies targeting faster dynamics or finer structures. Comparing gastric volume or motility measurements derived from the proposed GI MRI protocol with other terminal or invasive reference methods [6, 7, 8, 9] in rodents would further validate its accuracy and robustness. Extending the method in rodent disease models, such as chemically induced gastroparesis, would demonstrate its utility for diagnosing and monitoring clinically relevant phenotypes. Each of these directions holds potential to refine the protocol and expand its scope, propelling MRI‐based motility assessment toward an indispensable standard in preclinical gastroenterology.

Direct translation of the acquisition protocol from rats to humans poses several challenges. The contrast agent, such as Gd‐DTPA, used in rats, might not be ideal for human ingestion due to potential safety concerns; instead, our prior work demonstrated the use of Mn^2+^‐enriched natural food as a safer alternative [21]. Respiratory gating, effective in anesthetized rats with regular breathing, may be less reliable in humans, particularly in those with irregular breathing patterns, potentially leading to missed motility events. In such cases, free‐breathing acquisitions combined with motion‐mitigating strategies, such as radial sampling, might offer a more robust solution in human studies.

This study has limitations. The use of anesthesia remains a challenge. Even mild anesthetics can reduce gastric contractility [9, 39], and different agents or doses may affect motility patterns differently [39, 46]. Translating the MRI protocol from anesthetized conditions to awake conditions requires caution and fine‐tuning [39]. Another limitation is that mixing gastric juice with Gd‐labeled test meal in vivo can complicate the differentiation between meal volume and secretions, potentially confounding exact measures of emptying. Further innovations could incorporate multi‐contrast approaches or T_1_/T_2_ mapping to parse out the role of secretions [22, 23], differentiating labeled meal content from unlabeled fluid in the lumen. Lastly, while we focus on dynamic movements of the stomach, intragastric pressure is not addressed in our current protocol, but it is worth investigating.

## 5 Conclusion

The protocol described here provides an accessible and standardized framework for real‐time MRI of rat gastric motility. Its combination of respiratory gating, saturation pulses, accelerated imaging, and semi‐solid contrast meal preparation overcomes many prior constraints, facilitating high‐fidelity visualization of gastric motor function. From pharmacological testing to disease characterization, the method paves the way for comprehensive, repeated, and quantitative studies of rodent gastric physiology that can inform and complement human research.

## Author Contributions

Xiaokai Wang: conception and design, data acquisition, data analysis and interpretation, drafting and revising the manuscript; Ulrich M. Scheven: conception and design, data acquisition, data analysis and interpretation, revising the manuscript; Zhongming Liu: conception and design, data analysis and interpretation, revising the manuscript. All authors review and approve the final version of the manuscript and agree to be accountable for the contributions.

## Supporting information


**TABLE S1:** Quantitative effects of R and ACS on image quality of retrospectively reconstructed images (mean±standard deviation).

**TABLE S2:** Quantifications of antral motility from ground truth reference images and other retrospectively reconstructed images given different R and ACS (mean±standard deviation).

**VIDEO S1:** A dynamic series of one slice. The video is sped up by 20 times its original imaging speed.

**VIDEO S2:** A dynamic series of maximum intensity projections along the slice dimension. The video is sped up by 20 times its original imaging speed.

**DATA S1:** Supporting information.

## Data Availability

The data that support the findings of this study are available from the corresponding author upon reasonable request.
