# Supplementary figures and images for: Dynamic Magnetic Resonance Imaging of Whole‐Stomach Motility in Rats
Source: NMR Biomed. 2025 Sep 9;38(10):e70138. doi: 10.1002/nbm.70138 (PMC12421216; doi:10.1002/nbm.70138)

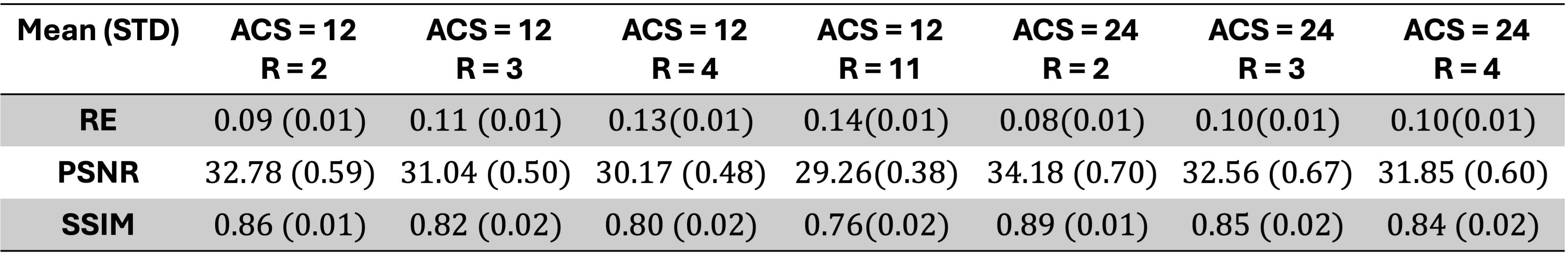

Supplement: Supplementary file 1 — TABLE S1: Quantitative effects of R and ACS on image quality of retrospectively reconstructed images (mean±standard deviation). [file NBM-38-e70138-s002.tif]

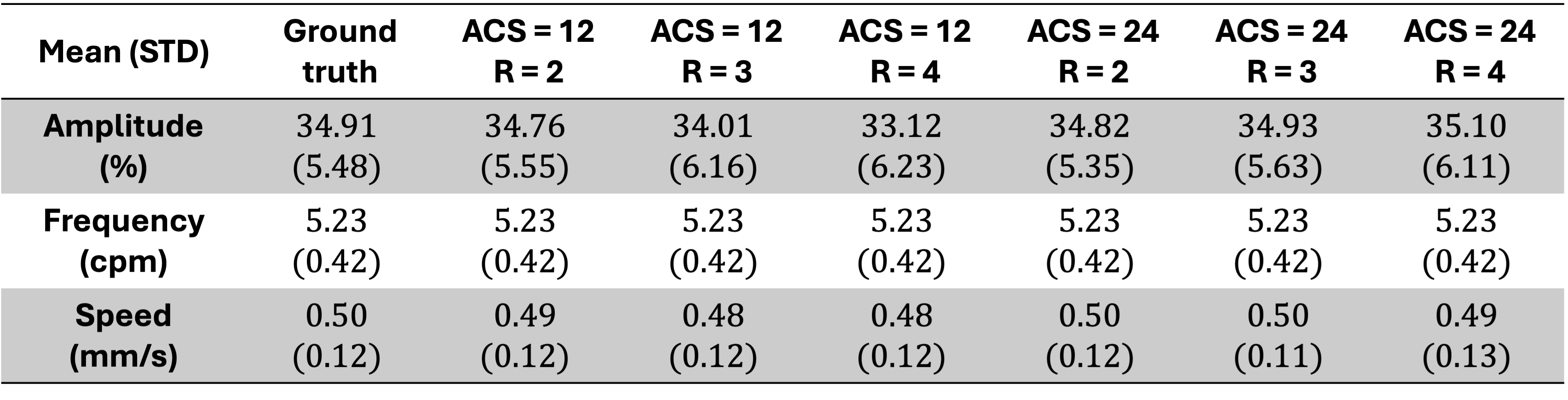

Supplement: Supplementary file 2 — TABLE S2: Quantifications of antral motility from ground truth reference images and other retrospectively reconstructed images given different R and ACS (mean±standard deviation). [file NBM-38-e70138-s005.png]

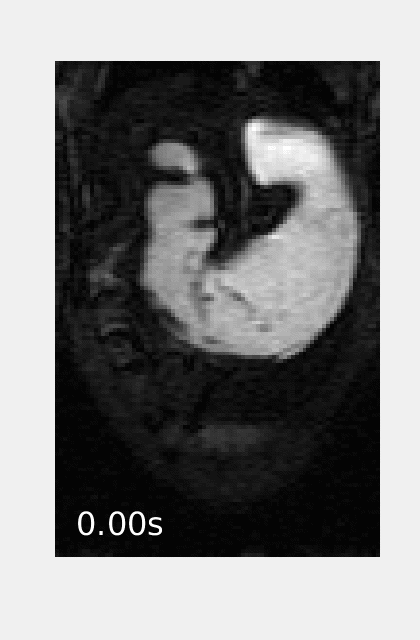

Supplement: Supplementary file 3 — VIDEO S1: A dynamic series of one slice. The video is sped up by 20 times its original imaging speed. [file NBM-38-e70138-s004.gif]

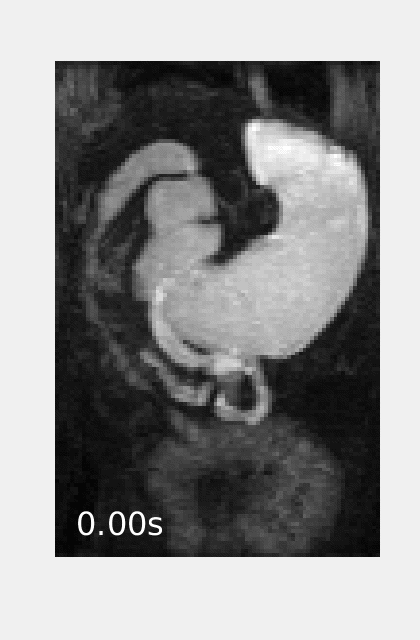

Supplement: Supplementary file 4 — VIDEO S2: A dynamic series of maximum intensity projections along the slice dimension. The video is sped up by 20 times its original imaging speed. [file NBM-38-e70138-s001.gif]
