# Supplementary material for: Dynamic Magnetic Resonance Imaging of Whole‐Stomach Motility in Rats
Source: NMR Biomed. 2025 Sep 9;38(10):e70138. doi: 10.1002/nbm.70138 (PMC12421216; doi:10.1002/nbm.70138)
Supplement: Supplementary file 5 — DATA S1: Supporting information. [file NBM-38-e70138-s003.docx]

### Supplementary Materials

# Supplementary tables

**Supplementary table 1.** Quantitative effects of R and ACS on image quality of retrospectively reconstructed images (mean$\pm$standard deviation).

**Supplementary table 2.** Quantifications of antral motility from ground truth reference images and other retrospectively reconstructed images given different R and ACS (mean$\pm$standard deviation).

**Supplementary video 1**. A dynamic series of one slice. The video is sped up by 20 times its original imaging speed.

**Supplementary video 2**. A dynamic series of maximum intensity projections along the slice dimension. The video is sped up by 20 times its original imaging speed.
